# Optical Properties of Silver-Mediated DNA from Molecular Dynamics and Time Dependent Density Functional Theory

**DOI:** 10.3390/ijms19082346

**Published:** 2018-08-09

**Authors:** Esko Makkonen, Patrick Rinke, Olga Lopez-Acevedo, Xi Chen

**Affiliations:** 1 Department of Applied Physics, Aalto University, P.O. Box 11100, 00076 Aalto, Finland; esko.makkonen@aalto.fi (E.M.); patrick.rinke@aalto.fi (P.R.); 2 Grupo de Física Atómica y Molecular, Instituto de Física, Facultad de Ciencias Exactas y Naturales, Universidad de Antioquia UdeA, Calle 70 No. 52-21, 050010 Medellín, Colombia

**Keywords:** DNA, silver, ECD, QM/MM, TDDFT

## Abstract

We report a combined quantum mechanics/molecular mechanics (QM/MM) molecular dynamics and time-dependent density functional (TDDFT) study of metal-mediated deoxyribonucleic acid (M-DNA) nanostructures. For the Ag${}^{+}$-mediated guanine tetramer, we found the maug-cc-pvdz basis set to be sufficient for calculating electronic circular dichroism (ECD) spectra. Our calculations further show that the B3LYP, CAM-B3LYP, B3LYP*, and PBE exchange-correlation functionals are all able to predict negative peaks in the measured ECD spectra within a 20 nm range. However, a spurious positive peak is present in the CAM-B3LYP ECD spectra. We trace the origins of this spurious peak and find that is likely due to the sensitivity of silver atoms to the amount of Hartree–Fock exchange in the exchange-correlation functional. Our presented approach provides guidance for future computational investigations of other Ag${}^{+}$-mediated DNA species.

## 1. Introduction

The nucleobases inside highly polymorphic deoxyribonucleic acid (DNA) molecules contain the essential genetic information for the creation and the functional properties of living organisms. In addition to hydrogen bonding, DNA is also capable of forming metallo base pairs in the presence of strongly bound metal ions. Interest in such metal-mediated DNA (M-DNA) nanostructures has rapidly grown due to their high potential for developing new materials for a wide range of biomedical and technological applications, such as bioimaging [1,2,3,4], metal ion detection [5,6,7], DNA sequencing [8], logic gates, and molecular devices [9]. Among the studied metal ions, Ag${}^{+}$ is particularly interesting, because it has low toxicity to humans and binds exclusively to the base pairs rather than the sugar–phosphate backbone. C-Ag${}^{+}$-C, G-Ag${}^{+}$-G, T-Ag${}^{+}$-T, A-Ag${}^{+}$-T (where A = adenine, C = cytosine, G = guanine, and T = thymine) and Ag${}^{+}$-mediated artificial DNA pairs have been reported [10,11,12]. In recent years, DNA-stabilized color-tunable fluorescent Ag clusters have also been synthesized, which contain both neutral Ag atoms and Ag${}^{+}$ ions, and are stabilized by Ag${}^{+}$-DNA interactions. They hold great potential for chemical and biochemical sensor applications and have attracted even more attention to the study of metal–DNA interactions [3,13].

Although the structures of some specific M-DNAs have been recently resolved via X-ray diffraction analysis [14,15,16], the atomic structures of M-DNAs are generally very difficult to obtain with X-ray crystallography, because M-DNA rarely crystallizes. Therefore, computational chemistry methods are important tools for deciphering the atomic structure of M-DNA and for interpreting the experimental spectroscopic data. In this context, electronic circular dichroism (ECD) spectroscopy is a versatile method to obtain structure information due to its high sensitivity to small structural changes in chiral optical active molecules, and a broad operational window in various solvent conditions. By comparing computed with measured ECD spectra, the structure of M-DNA can be inferred. This approach has been used for mapping the conformal properties of various DNA-based systems including M-DNAs [17,18,19]. Additionally, ECD provides a reliable benchmark method for validating computational approaches, due to its structural sensitivity.

However, modeling the ECD spectra of M-DNAs remains a difficult task. Since the prepared materials exist mostly in aqueous solution, the effect of the environment on the structures and properties have to be taken into account. This leads to an increased system size, which is prohibitive for a full quantum level calculation, and thus requires multi-scale strategies, i.e., hybrid quantum mechanics/molecular mechanics (QM/MM) methods. In order to compare with experiments, the structures for simulating ECD need to be chosen carefully from QM/MM simulations, bearing in mind that they exhibit the key structural properties of the system but are still computationally affordable. Last, as always with density functional theory (DFT), the used exchange-correlation functional has to be suitable for the system under study. It is well known that hybrid or long-range corrected hybrid functionals are usually more accurate for studying organic molecule, whereas the GGA functionals are commonly used for bulk metal and solid state systems. Currently, it is not fully conclusive how to choose the optimal exchange-correlation functional for M-DNA systems.

In this work, we chose to study Ag${}^{+}$-mediated guanine tetramer, whose ECD has been reported recently [20] to systematically study how the basis sets, the exchange-correlation functionals, and the implicit water affect simulations of the ECD. This work will provide some useful knowledge to choose suitable computational methods for studying silver-DNA systems and other M-DNAs.

## 2. Results and Discussion

### 2.1. QM/MM Molecular Dynamics and the Atomic Structures of the Ag${}^{+}$-Mediated Guanine Tetramer

To unveil the atomic structure of the Ag${}^{+}$-mediated guanine duplex in water, we have previously performed QM/MM molecular dynamics calculations for the smallest Ag${}^{+}$-mediated guanine duplex G${}_{2}$-Ag${}_{2}^{+}$-G${}_{2}$, namely the Ag${}^{+}$-mediated guanine tetramer [21]. Silver and DNA atoms were treated at the DFT level. Solvation was modeled with a 4 nm box of classical water molecules and the Amber force field [22]. The calculations were carried out with the CP2K code [23], and the Perdew–Burke–Ernzerhof (PBE) exchange and correlation potential [24]. Grimme’s D3 dispersive corrections were used to account for long-range van der Waals interactions [25]. Before the QM/MM simulations, the system was first equilibrated at 300 K with a classical NVT run of 2 ns, using the Amber force field. Then the QM/MM simulations were performed for 21 ps with a time-step of 0.5 ps. In the QM/MM simulations, van de Waals interactions between the QM and MM regions were approximated by classical force fields. Electrostatic interactions between the two subsystems were accounted for using an embedding approach, where point charges located at the water molecules (MM region) can polarize the Ag${}^{+}$-mediated DNA (QM region). By comparing the root mean square distance of the heavy atoms to the initial structure positions, we can conclude that the system reached an equilibrium regime after 16 ps. Therefore, we used only the last 5 ps of the trajectory for analysis. Further details of these simulations can be found in Reference [21].

**Figure 1:**
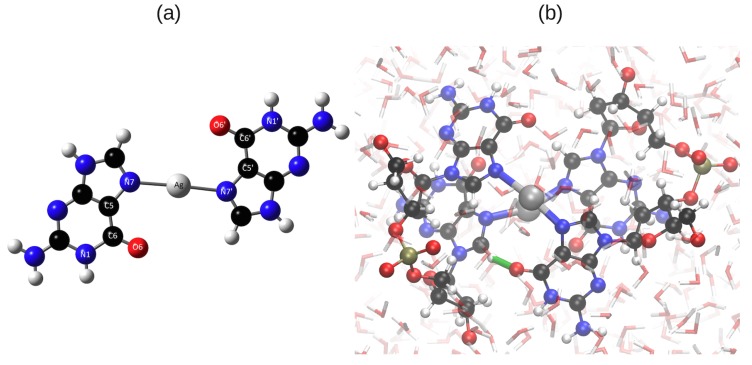
(**a**) Ag${}^{+}$ binds to N7 and N7’ in the Hoogsteen region; (**b**) One typical structure of G${}_{2}$-Ag${}_{2}^{+}$-G${}_{2}$ from the final 0.5 ps of the hybrid quantum mechanics/molecular mechanics (QM/MM) simulation. The structure was taken from our previous work [21] and replotted here. The interplanar H-bond is highlighted with a green line. Atomic color code: Ag: silver, N: blue, C: black, O: red, P: Olive and H: white.

In this work we adopted the structures for the ECD analysis from our previous QM/MM study. We briefly recap the structural findings from Reference [21]. In brief, Ag${}^{+}$ ions bind to the N atoms in the Hoogsteen region (Figure 1a) and the DNA forms a left-handed helix. The Ag–Ag distance oscillates around an average value of 3.45 Å with a standard deviation of 0.27 Å. A typical structure of an Ag${}^{+}$-mediated tetramer is shown in Figure 1b.

### 2.2. Time-Dependent Density Functional Theory Study for the Optical Properties of the Ag${}^{+}$-Mediated Guanine Tetramer

Previously we have simulated the ECD of G${}_{2}$-Ag${}_{2}^{+}$-G${}_{2}$ by linear-response time-dependent density functional theory (TDDFT) implemented in GPAW [26,27]. GPAW is a DFT code based on the projector-augmented wave (PAW) method and the wave functions are expanded on uniform real-space grids. We used the LB94 [28] exchange-correlation functional in that simulation. Although the simulated average ECD and the measured ECD of G${}_{2}$-Ag${}_{2}^{+}$-G${}_{2}$ were in satisfactory agreement [21], it is worthwhile to check how the basis sets and the exchange-correlation functional affect the ECD simulations when we switch to the Gaussian16 code [29]. One particular question we are interested in is, can hybrid functionals improve the agreement between the calculated and the measured ECD?

In this work, we employed the Gaussian16 code [29] to simulate the ECD spectra. The details can be found in the Materials and Methods section.

#### 2.2.1. Experimental ECD Spectra of Ag${}^{+}$-Mediated Guanine Species

**Figure 2:**
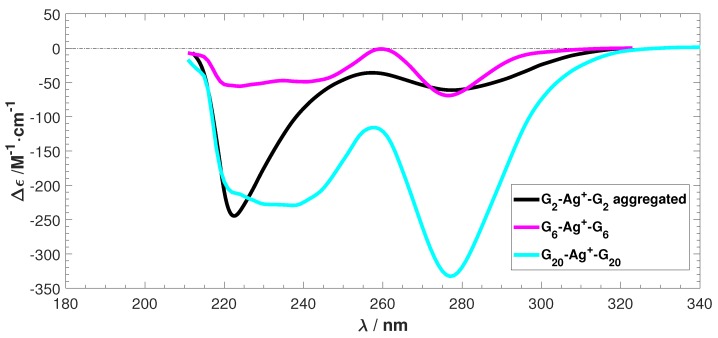
The experimental electronic circular dichroism (ECD) spectra of Ag${}^{+}$ stabilized G${}_{2}$, G${}_{6}$, and G${}_{20}$. Δϵ is differential molar extinction coefficients Δϵ = $\epsilon_{L}$−$\epsilon_{R}$, λ is the wavelength. Data reproduced with permission from Swasey, S. and Gwinn, E. *New J. Phys.* **2016**, *18*, 045008–045025. [20].

The experimental ECD spectra for three Ag${}^{+}$-mediated guanine species obtained from Reference [20] are shown in Figure 2. The spectra of G${}_{6}$-Ag${}_{6}^{+}$-G${}_{6}$ and G${}_{20}$-Ag${}_{20}^{+}$-G${}_{20}$ exhibit similar features, but the G${}_{2}$-Ag${}_{2}^{+}$-G${}_{2}$ spectrum differs. This is possibly due to aggregation induced by the Ag${}^{+}$ ions, which prevents a comparison of the intensity between the predicted and the experimental ECD [20,30,31]. However, G${}_{2}$-Ag${}_{2}^{+}$-G${}_{2}$ presents a good model system to study the ECD of Ag${}^{+}$-mediated guanine duplexes, especially in terms of the negative peak locations and the absence of any positive peaks.

#### 2.2.2. Tests for Basis Set Convergence

We first tested the basis set convergence on a single sampled structure. Two popular hybrid exchange-correlation functionals, B3LYP [32,33] and the Coulomb-attenuated B3LYP (CAM-B3LYP) [34], were used for the tests. A single compute node with 20 Haswell cores and 40 GB of shared memory was used. To assure convergence in the range of 180–360 nm, 300 and 150 excited singlet states were used in the B3LYP and CAM-B3LYP calculations, respectively.

**Figure 3:**
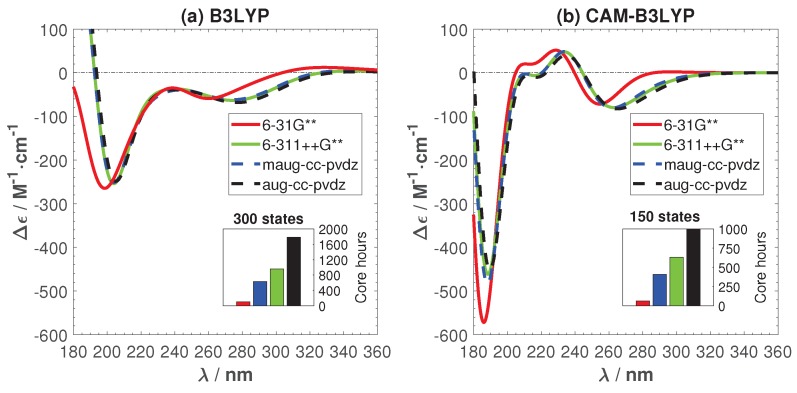
(main figures) The effects of basis sets and exchange correlation-functional on the ECD spectrum of a single snapshot structure and (inset figures) the related cpu time costs. (**a**) B3LYP; (**b**) CAM-B3LYP.

Figure 3 shows the effects of employed basis sets for C, H, N, and O atoms and the related cost for calculating the ECD spectrum of a single sampled structure. For Ag, the LANL2DZ/ECP basis set was used in all calculations. Figure 3 shows that B3LYP is more sensitive to the basis sets when diffusion functions are added. For both functionals, basis set convergence is reached by the minimally augmented Dunning basis set, maug-cc-pvdz. It gives practically the same ECD spectra as the aug-cc-pvdz and 6-311++G**, which have been previously used for M-DNAs and pure base pair systems [19,35,36] with good performance. The capability of maug-cc-pvdz to match aug-cc-pvdz has been shown previously for other systems and similar to this study, doing so with much lower computational cost [37]. Based on the results presented in Figure 3, maug-cc-pvdz was chosen for the remainder of this work.

The two functionals give significantly different ECD spectra. CAM-B3LYP exhibits two peculiar positive peaks at 204 and 234 nm that are absent in the B3LYP spectrum. In the following, we will trace the origin of this difference and try to find the best functionals for studying the optical properties of Ag${}^{+}$-DNA.

#### 2.2.3. Calculated Average ECD and Comparison to the Experimental ECD

Previous work [21] has highlighted the importance of sampling molecular dynamics trajectories and comparing averaged ECD spectra to experiment. Typically ten sampled structures were found to be adequate for G${}_{2}$-Ag${}_{2}^{+}$-G${}_{2}$ in water. Therefore, in this section, we will discuss the calculated average ECD spectra and compare them to the experimental ECD in Figure 2.

**Figure 4:**
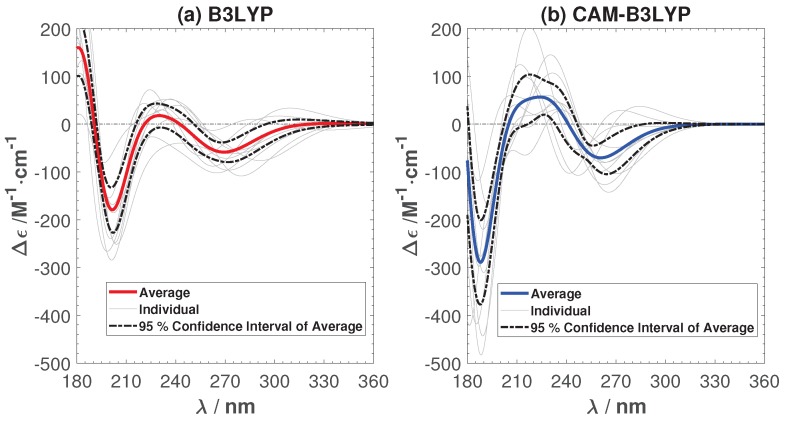
The average and individual ECD spectra of ten sampled structures with B3LYP (**a**) and CAM-B3LYP (**b**). Spectra are not shifted, but presented as calculated.

Figure 4 shows the calculated average and individual ECD with the B3LYP and CAM-B3LYP functionals, respectively. In order to show that ten samples are enough to capture the main features of the ECD, we have also added the 95% confidence interval of the average ECD in the figure. The negative peaks predicted by B3YLP lie around 200 and 270 nm, while the CAM-B3YLP peaks are around 190 and 260 nm. They are both shifted towards the short wavelength region compared to experiment (peaks around 220 and 280 nm). The B3LYP ECD is in good agreement with the experimental ECD spectrum. The main difference is a small shoulder (<20 M${}^{-1}$cm${}^{-1}$) between 220 and 240 nm. This shoulder becomes much more pronounced in CAM-B3YLP (about 50 M${}^{-1}$cm${}^{-1}$). Such a shoulder, or in fact any positive peak, is not present in the experimental ECD spectra. This poses the question of whether CAM-B3YLP predicts artifacts for our system. To make sure the peculiar positive feature in the CAM-B3LYP spectra is not due to the solution, we have calculated the ECD in an implicit solution environment. The positive peak remains. More details can be found in the Appendix A.

In the following, we will investigate different reasons for the difference between B3LYP and CAM-B3LYP and for CAM-B3LYP’s erroneous behavior.

#### 2.2.4. Amount of Hartree–Fock Exchange

The amount of Hartree–Fock (HF) exchange in hybrid functionals is known to be crucial in studying transition metal complexes [38,39], and lowering the amount of HF exchange in B3LYP from 20% to 15% has proven to be a valid modification to correctly obtain energetics for proton and electron transfer reactions in comparison with experimental values [38,39,40,41]. We have also tested this modified B3LYP, called B3LYP*, for the Ag${}^{+}$-mediated guanine tetramer, along with a pure GGA functional PBE, which has no HF exchange.

**Figure 5:**
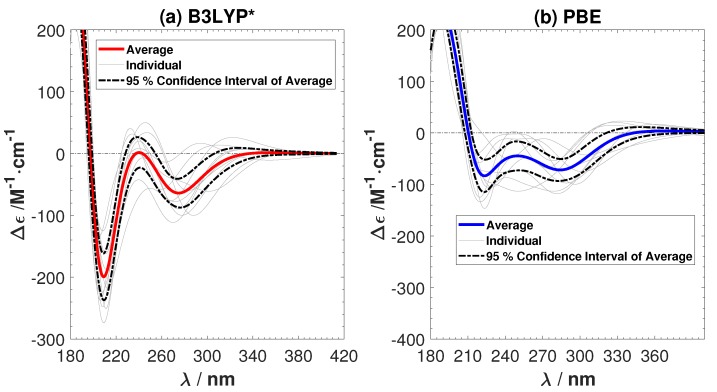
The average and individual ECD spectra of ten sampled structures with (**a**) the B3LYP* (15% Hartree–Fock (HF) exchange) and (**b**) the Perdew–Burke–Ernzerhof (PBE) functionals. No shiftings of the calculated spectra are done.

The averaged ECD spectra from ten sampled B3LYP* and PBE structures are shown in Figure 5. The ECD of the individual samples and the 95% confidence interval of the average ECD are given in the same figure. The amount of HF exchange has a clear effect, since the positive shoulder has now vanished. Both B3LYP* and PBE successfully predict the two negative peaks in the experimental ECD. B3LYP* gives a better agreement with the intensity of the peaks in the experimental ECD of G${}_{2}$-Ag${}_{2}^{+}$-G${}_{2}$, but PBE predicts the positions of the peaks more accurately. There are spurious peaks in the individual ECD spectra predicted by PBE in the long wavelength region. However they cancel each other out in the averaged ECD spectrum. Comparing the ECD spectra from B3YLP, CAM-B3YLP, B3YLP*, and PBE, we conclude that it is important to adjust the amount of Hartree–Fock exchange in exchange-correlation functional, in order to predict the ECD of G${}_{2}$-Ag${}^{+}$-G${}_{2}$ accurately. The sensitivity to the amount of exact exchange might be a common issue for other M-DNA systems, especially for the Ag${}^{+}$-mediated guanine-rich DNA systems.

#### 2.2.5. Effect of the Long-Range Correction in CAM-B3LYP

B3LYP adequately predicts ECD spectra for many organic molecules [42,43]. However, CAM-B3LYP is a functional built to overcome the deficiencies of B3LYP and to correctly describe charge transfer, local excitations, and Rydberg excitations [34]. It has been assessed for a broad set of small main group and organic molecules [44], and has been demonstrated to outperform B3LYP for chiral alkenes [45], chiral aromatic nitro compounds [46], and metal-porphyrin complexes [47]. It is very interesting to understand why for our Ag${}^{+}$-guanine system, CAM-B3YLP gives less accurate ECD spectra than B3LYP. For this purpose, we first tested the two functionals on two DNA systems without metal ions: one is a C${}_{2}$-G${}_{2}$ tetramer taken from a reported B-DNA structure (9BNA in the protein data bank), and another is a G${}_{2}$-G${}_{2}$ tetramer taken from the same sample used in Figure 3, in which the Ag atoms were deleted.

**Figure 6:**
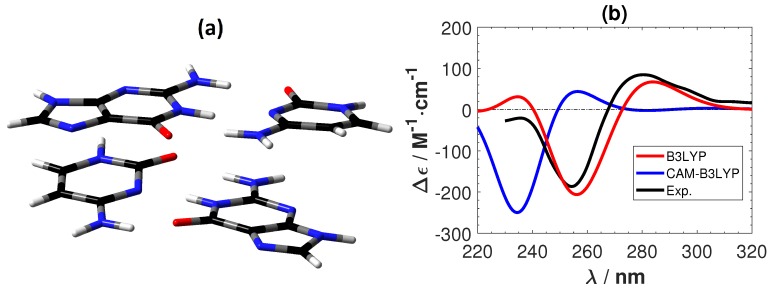
(**a**) The structure of a plain B-DNA tetramer. The color of atoms : O: red, C: black, N: blue and H: white. (**b**) The corresponding calculated and experimental [48,49] ECD spectra. No shifting of the calculated spectra are done.

**Figure 7:**
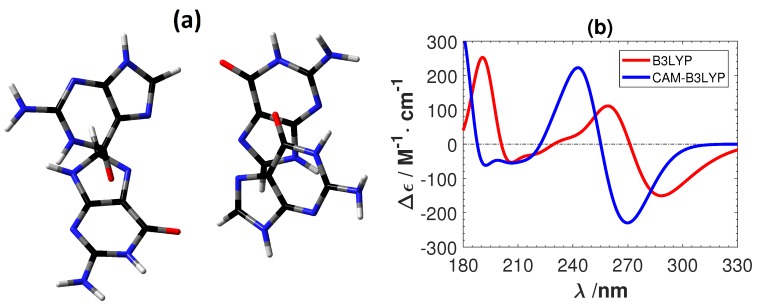
(**a**) The structure of a plain guanine tetramer. The color of atoms : O: red, C: black, N: blue and H: white. (**b**) The corresponding calculated ECD spectra. No shifting of the calculated spectra are done.

Figure 6 shows the experimental ECD of a B-DNA [48,49] (d(m5C-G-C-G-m5C-G)) and the calculated ECD spectra of the C${}_{2}$-G${}_{2}$ system using B3LYP and CAM-B3LYP. As for the Ag${}^{+}$-mediated guanine tetramer we removed the sugar–phosphate backbone and optimized the replacement hydrogen atoms at the B3LYP/6-31G** level. B3LYP predicts the correct positions and intensity of the peaks in the experimental ECD. On the other hand, CAM-B3LYP also gives overall correct features, but with a shift of roughly 20 nm towards the lower wavelength region. A shift of the same order (roughly 20 nm) was also obtained between CAM-B3LYP and B3LYP for G${}_{2}$-G${}_{2}$ as shown in Figure 7. Although CAM-B3LYP predicts more intensive peaks with shifts to lower wavelengths, these two functionals give qualitatively the same ECD features for the two DNA structures without metal ions.

To further investigate the difference between B3LYP and CAM-B3LYP in our system, we calculated the ECD spectra of a sampled structure by gradually changing CAM-B3LYP to B3LYP. This was done by changing the short-range parameter α and the long-range parameter β in the definition of Coulomb-attenuation range $r_{12}$ [34]:

(1) $$\frac{1}{r_{12}}=\frac{1-[\alpha +\beta \cdot \mathrm{erf}\left(\mu r_{12}\right)]}{r_{12}}+\frac{\alpha +\beta \cdot \mathrm{erf}(\mu r_{12})}{r_{12}}.$$

The HF exchange has a weight of α at $r_{12}=0$ and α+β at $r_{12}=\infty$. Correspondingly, DFT exchange is 1−α at $r_{12}=0$ and 1−(α+β) at $r_{12}=\infty$. The default parameter values for CAM-B3LYP are α=0.19,β=0.46, and μ=0.33. The parameter μ sets the midpoint behavior, and was kept at the default value of 0.33. With parameters α=0.2 and β=0, B3LYP is obtained.

**Figure 8:**
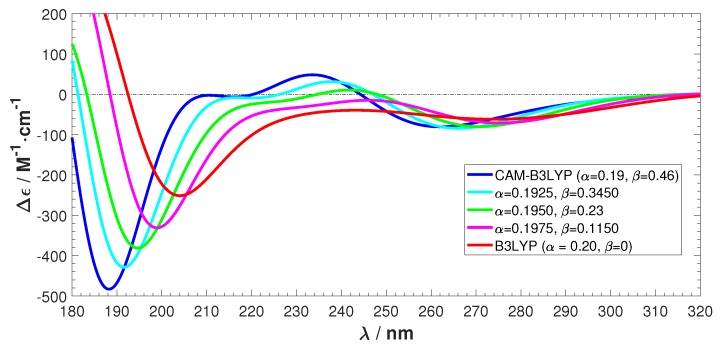
ECD spectra transformation from CAM-B3LYP/maug-cc-pvdz to B3LYP/maug-cc-pvdz.

**Figure 9:**
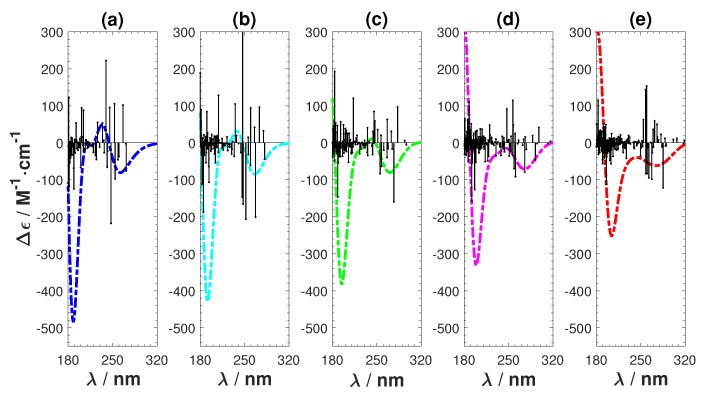
Transformation from CAM-B3LYP to B3LYP by changing the short- and long-range parameters. (**a**) CAM-B3LYP (α=0.19,β=0.46); (**b**) α=0.1925,β=0.3450; (**c**) α=0.1950,β=0.23; (**d**) α=0.1975,β=0.1150; and (**e**) B3LYP (α=0.2,β=0).

The results are shown in Figure 8 and Figure 9. Figure 8 shows that we achieve a smooth transformation from CAM-B3LYP to B3LYP. The absolute intensity of the negative peak in the short wavelength region reduces and the ECD spectrum shifts towards the long wavelength direction. The positive features observed in CAM-B3LYP smoothly vanish. Figure 9 also shows the transformation, together with the involved rotatory strengths of the singlet excitations. The positive peak emerges from a collection of high intensity excitations, which are gradually shifting towards the higher wavelength region. From these tests, we can conclude that applying the long-range corrected hybrid function CAM-B3LYP to Ag atoms cause the unnatural features in the calculated ECD.

**Figure 10:**
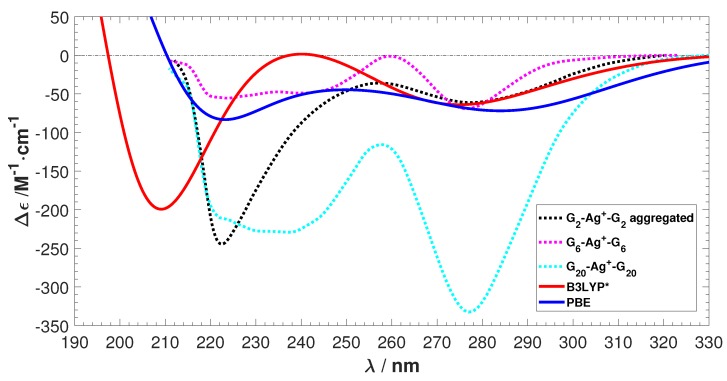
The experimental ECD [20] spectra shown along with our best prediction by B3LYP*. No shiftings of spectra are done.

Finally, to show the capability of our methodology to predict ECD spectra, our best predictions without any fitting are shown alongside experimental spectra in Figure 10. The two negative peaks are predicted within 20 nm.

## 3. Materials and Methods

In this work, we employed the Gaussian16 code [29] which uses Gaussian type local basis sets to simulate the ECD spectra. The sugar–phosphate backbones were replaced by hydrogen atoms to make the computational cost feasible. We argue that this is reasonable, because the sugar–phosphate backbone is not the main interest of our study and does not affect the optical properties in the region of interest (long distances from the center Ag${}^{+}$-ions and transitions at high energies [50]). The replacement hydrogen atoms were then relaxed at the B3LYP/6-31G** level, while keeping the rest of the atoms frozen.

Singlet transitions were included in excited state calculation so that the high-energy wavelength region up to 170 nm was covered (to assure convergence up to 180 nm) for the Ag${}^{+}$-mediated tetramer and guanine tetramer. The first 300, 500, 700, and 150 transitions were included for B3LYP, B3LYP*, PBE, and CAM-B3LYP calculations, respectively. For B-DNA, the high-energy wavelength region, up to 210 nm, was covered by the first 60 and 80 transitions for CAM-B3LYP and B3LYP, respectively.

The following relations were used in the ECD spectra simulations [51,52,53]:

(2) $$\Delta \epsilon =4a\sum_{n}R_{n}E_{n}\sigma_{n}\left(E\right)$$

(3) $$a=\frac{4\pi N_{A}}{3\ln\left(10\right)10^{3}}\frac{2\pi }{hc}$$

(4) $$\sigma_{n}\left(E\right)=\frac{1}{\sqrt{2\pi }\sigma }\exp\left(-\frac{1}{2\sigma^{2}}{\left(E-E_{n}\right)}^{2}\right),$$

where Δϵ is molar circular dichroism (in M${}^{-1}$cm${}^{-1}$), $N_{A}$ is Avogagro’s constant (in mol${}^{-1}$), *h* is Planck’s constant (in Js), *c* is the speed of light (in cm s${}^{-1}$), $R_{n}$ is length-gauge rotatory strength (in 10${}^{-40}$ cgs), *E* is the energy of the incident light (in eV), $E_{n}$ is the excitation energy to state *n* (in eV), and σ is the exponential half-width in Gaussian convolution (here 0.3 eV was used for the Ag${}^{+}$-mediated tetramer and 0.2 eV for the B-DNA and plain guanine tetramers).

## 4. Conclusions

In summary, we have used TDDFT and QM/MM to study the ECD spectra of the Ag${}^{+}$-mediated guanine tetramer. We have discussed how the basis sets and the exchange-correlation functional affect the computed ECD spectra. For the basis sets, convergence is achieved at the maug-cc-pvdz level. The tested functionals CAM-B3LYP, B3LYP, B3LYP* (15% HF), and PBE all successfully predict two negative peaks within a 20 nm error. B3LYP* and PBE give the best agreement of the measured ECD. Conversely, CAM-B3LYP produces a spurious positive feature, which is not observed in experiment. Our study highlights the importance of the exchange-correlation functional. In future ECD studies of metal enhanced DNA in water, we recommend to start with the PBE or B3LYP* functionals. Due to the much lower computational cost and satisfactory accuracy of PBE, it is a better choice for large M-DNA systems.

## Figures and Tables

**Figure A1:**
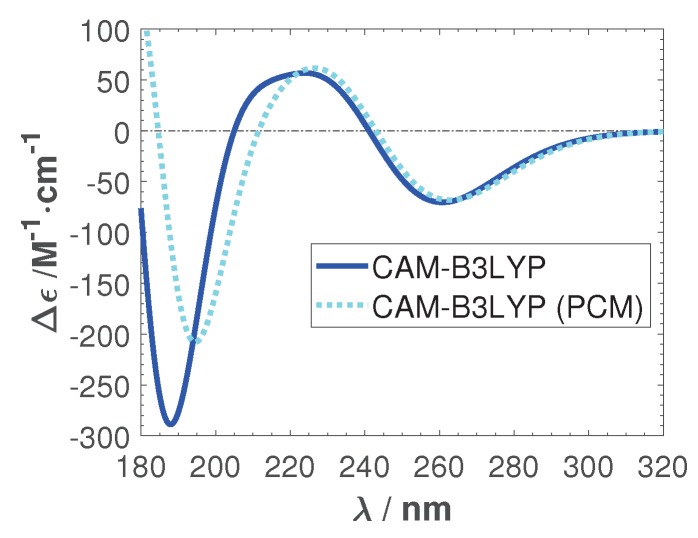
The average ECD spectra of ten sampled structures with CAM-B3LYP in the gas phase and in a polarizable continuum model (PCM) water environment. No calculated spectra were shifted.

## Appendix A. Solvent Effect on the CAM-B3LYP Calculated ECD

To investigate if the peculiar positive feature in the CAM-B3LYP spectra is due to the solution, we calculate the ECD in a solvent environment. We modeled water implicitly by a polarizable continuum model (PCM) [54]. Here we used a linear-response procedure, where the solvent effect on the self-consistent field (SCF), density of solute, is computed by adding the required terms to the TDDFT equations [55]. Figure A1 shows the effects of the implicit solvent model on the average ECD of ten sampled structures. The positive peak remains.
